# Heavy metal bioremediation using microbially induced carbonate precipitation: Key factors and enhancement strategies

**DOI:** 10.3389/fmicb.2023.1116970

**Published:** 2023-02-02

**Authors:** Wenchao Zhang, Hong Zhang, Ruyue Xu, Haichen Qin, Hengwei Liu, Kun Zhao

**Affiliations:** ^1^School of Chemistry and Life Sciences, Suzhou University of Science and Technology, Suzhou, China; ^2^Frontiers Science Center for Synthetic Biology, Key Laboratory of Systems Bioengineering (Ministry of Education), School of Chemical Engineering and Technology, Tianjin University, Tianjin, China; ^3^Sichuan Provincial People’s Hospital, University of Electronic Science and Technology of China, Chengdu, China; ^4^Insitute of Fundamental and Frontier Sciences, University of Electronic Science and Technology of China, Chengdu, China

**Keywords:** microbially induced carbonate precipitation, ureolytic bacteria, heavy metal, immobilization efficiency, enhancement strategies

## Abstract

With the development of economy, heavy metal (HM) contamination has become an issue of global concern, seriously threating animal and human health. Looking for appropriate methods that decrease their bioavailability in the environment is crucial. Microbially induced carbonate precipitation (MICP) has been proposed as a promising bioremediation method to immobilize contaminating metals in a sustainable, eco-friendly, and energy saving manner. However, its performance is always affected by many factors in practical application, both intrinsic and external. This paper mainly introduced ureolytic bacteria-induced carbonate precipitation and its implements in HM bioremediation. The mechanism of HM immobilization and *in-situ* application strategies (that is, biostimulation and bioaugmentation) of MICP are briefly discussed. The bacterial strains, culture media, as well as HMs characteristics, pH and temperature, etc. are all critical factors that control the success of MICP in HM bioremediation. The survivability and tolerance of ureolytic bacteria under harsh conditions, especially in HM contaminated areas, have been a bottleneck for an effective application of MICP in bioremediation. The effective strategies for enhancing tolerance of bacteria to HMs and improving the MICP performance were categorized to provide an in-depth overview of various biotechnological approaches. Finally, the technical barriers and future outlook are discussed. This review may provide insights into controlling MICP treatment technique for further field applications, in order to enable better control and performance in the complex and ever-changing environmental systems.

## 1. Introduction

Due to urbanization and excessive anthropogenic activities, heavy metal (HM) pollution in diverse ecosystems has emerged as a major global concern in recent years. These pollutants critically threaten the stability of ecosystems and the health of humans owing to their non-biodegradability and bioaccumulation *via* the food chain (Pratush et al., 2018; Yin et al., 2019; Haque et al., 2023). Even at extremely low doses, the exposure of various HMs such as lead is poisonous, causing cell toxicity, mutation, and cancer (García-Lestón et al., 2010). Consequently, HM pollution has become one of the most serious environmental issues, and pollution remediation is crucial.

Various physicochemical approaches have been developed to remediate HMs, including but not limited to chemical precipitation, adsorption, ion exchange, membrane separation and electrochemical technique (Ayangbenro and Babalola, 2017; Azimi et al., 2017; Rahman, 2020), which are typically expensive, energy-intensive, and ineffective. In contrast, biomineralization-based remediation is eco-friendlier and more cost-effective; consequently, it is gradually gaining acceptance as a technology for HM remediation (Hu et al., 2021; Oliveira et al., 2021; Zeng et al., 2021; Xue et al., 2022). Biomineralization is the process that microbial metabolic activity modifies the chemical environment to promote the formation of mineralized structures (Stocks-Fischer et al., 1999; Qin et al., 2020). It is a natural, pervasive phenomenon that is capable of producing a wide variety of minerals (over 60 types) by microbes. Presently, scholars from across the globe are focusing on harnessing the technological uses of these biominerals in a range of aspects. Microbially induced carbonate precipitation (MICP) is the most extensively researched branch of biomineralization, which holds promise for diverse fields ranging from biomedicine [i.e., bone regeneration (Li et al., 2021a)], geotechnology (i.e., soil stabilization, pollutant remediation (Ashraf et al., 2017; Yin et al., 2021b)), to civil engineering (i.e., bioconcrete (Castro-Alonso et al., 2019)). Numerous microbial processes, such as ureolysis, photosynthesis, denitrification, ammonification, sulfate reduction, and methane oxidation, have been found to contribute to the formation of carbonate precipitation (Zhu and Dittrich, 2016; Lee and Park, 2018; Jain et al., 2021). Among these metabolic pathways, carbonate production mediated by ureolysis has been found to be the most energy-efficient, simple, widespread, and calcification-prone, as well as the easiest to control (Wang et al., 2016; Seifan and Berenjian, 2019; Liu et al., 2021). Numerous studies have examined the bioremediation potential of microbes at HM-contaminated sites by inducing carbonate precipitation and transforming free toxic ions into inactive forms in stable and long-lasting matrices (Achal et al., 2011; Fang et al., 2021; Yin et al., 2021a; Li et al., 2021b). Therefore, it is widely accepted that MICP is an environmentally friendly method for accelerating HM removal rates by optimizing limiting factors (Wang et al., 2021; Li et al., 2022). MICP is a dynamically changing process that engages in minerals crystallization evolution and microbial metabolism, controlled by multiple factors in complex environments, including pH, temperature, bacterial strains, availability of nutrients, calcium sources, etc. (Rahman and Singh, 2020; Supekar et al., 2020; Tang et al., 2020; Song et al., 2022).

Unfortunately, the immobilization rate of HMs decreases with increasing contaminant concentration (Oliveira et al., 2021) and varies based on the bioavailability of contaminants and the characteristics of the contaminated area, particularly the nature of HM-tolerant microorganisms. The toxicity of HMs typically inhibits microbial activity, which slows the bioremediation process and reduces the overall efficiency. Several bioremediation strategies have been employed to enhance microbial activity during MICP-based HM remediation (Hu et al., 2021; Oliveira et al., 2021; Song et al., 2021). However, adequate summary information regarding improved bioremediation is not yet available. This review focuses on the various limiting factors that impact the performance of MICP and the bioremediation strategies for improving microbial tolerance to HMs. This will contribute to a deeper understanding of MICP mechanisms, the discovery of more effective bioremediation strategies, the development of the next generation of bioremediation technology, and a better grasp of its field-scale applications. We anticipate that our discussion can be extended to the study of numerous other microbe-mineral systems in addition to ureolytic MICP.

## 2. HMs removal mechanism by ureolysis-driven MICP

**Figure 1:**
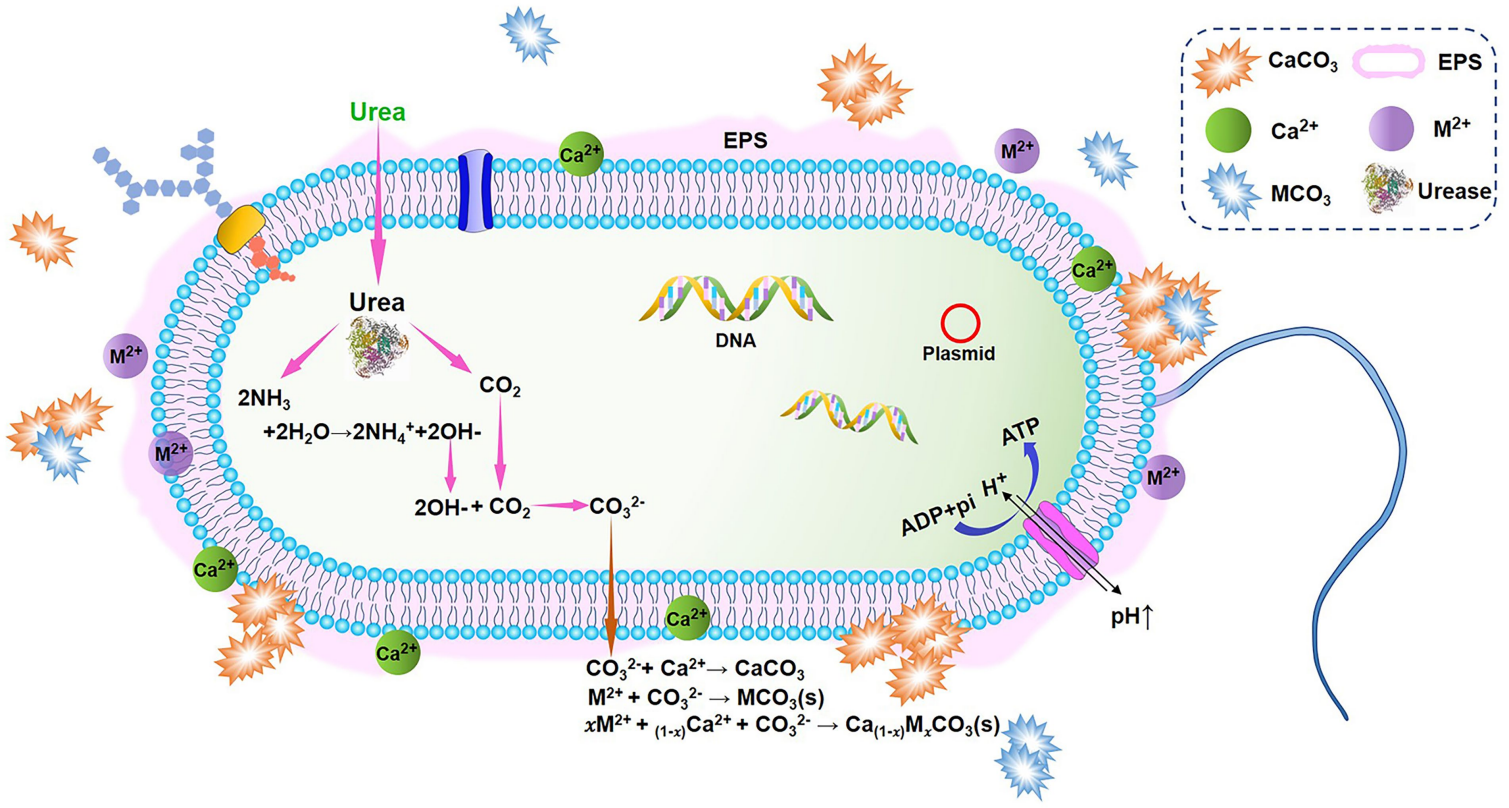
A schematic diagram of microbially induced carbonate precipitation (MICP) based on urea hydrolysis. Modified from Song et al. (2022).

The simplest and most easily controlled mechanism of MICP, ureolytic microorganism-induced carbonate precipitation has the potential to produce large quantities of carbonates in a brief period (in the rest of the paper, MICP generally refers to ureolytic MICP for simplicity). This pathway employs microorganisms that secrete a large amount of highly reactive urease to catalyze the hydrolysis of urea, resulting in the production of CO_2_ and NH_3_ (Equation 1). Subsequently, these products equilibrate in the solution to form bicarbonate, ammonium, and hydroxide (Equations 2, 3), leading to an increase in pH (alkalinity) and the formation of carbonate ions (DeJong et al., 2010; Dhami et al., 2013; Zhang et al., 2018; Equation 3). The carbonate can precipitate out of solution in the presence of divalent cations at sufficient ionic activity (Equation 4). Figure 1 illustrates a schematic diagram of MICP. The equation for the reaction is as follows:

(1) $$\mathrm{CO}{\left({\mathrm{NH}}_{2}\right)}_{2}+H_{2}O\overset{\mathrm{Urease}}{\to }\;{\mathrm{2NH}}_{3}+{\mathrm{CO}}_{2}$$

(2) $${\mathrm{2NH}}_{3}+{\mathrm{2H}}_{2}O\;\to {\mathrm{2NH}}_{4}{}^{+}+{\mathrm{2OH}}^{-}$$

(3) $${\mathrm{CO}}_{2}+{\mathrm{2OH}}^{-}\;\to {\mathrm{HCO}}_{3}{}^{-}+{\mathrm{OH}}^{-}\to \;{\mathrm{CO}}_{3}{}^{2-}+H_{2}O$$

(4) $${\mathrm{Ca}}^{2+}+{\mathrm{CO}}_{3}{}^{2-}\to \;{\mathrm{CaCO}}_{3}\left(s\right)$$

(5) $$M^{2+}+{\mathrm{CO}}_{3}{}^{2-}\to \;{\mathrm{MCO}}_{3}\left(s\right)$$

(6) $$xM^{2+}+\left(1-x\right)\;{\mathrm{Ca}}^{2+}+{\mathrm{CO}}_{3}{}^{2-}\to \;{\mathrm{Ca}}_{\left(1-x\right)}M_{x}{\mathrm{CO}}_{3}\left(s\right)$$

Other metals and radionuclides, like cadmium (Yin et al., 2021a) and strontium (Lauchnor et al., 2013) can be precipitated to form their own insoluble carbonate minerals *via* the same route (Equation 5, M^2+^ represents a divalent metal cation). Alternatively, they can be co-precipitated with calcium carbonate (Equation 6) if calcifying microbes can survive in toxic metal environments. HM ions with ion radii close to that of Ca^2+^ (1.0 Å; e.g., Cu^2+^, Cd^2+^, Pb^2+^, Zn^2+^, and Sr^2+^) can be incorporated in CaCO_3_ crystal lattice by isomorphic substitution of Ca^2+^ or by penetrating the interstice or defect of crystal (Pan, 2009; Achal et al., 2011; Jain and Arnepalli, 2019; Moghal et al., 2020; Kim et al., 2021). After that, HMs are changed from soluble ions to insoluble forms, preventing themselves from being released back into the environment. The pervasiveness of MICP and its ability to trap HMs make it a viable *in-situ* remediation method for contaminated sites. In addition, its insensitivity to redox potential changes in surroundings makes it highly effective and keeps long-term stability in bioremediation.

## 3. Key factors influencing the performance of MICP bioremediation

**Figure 2:**
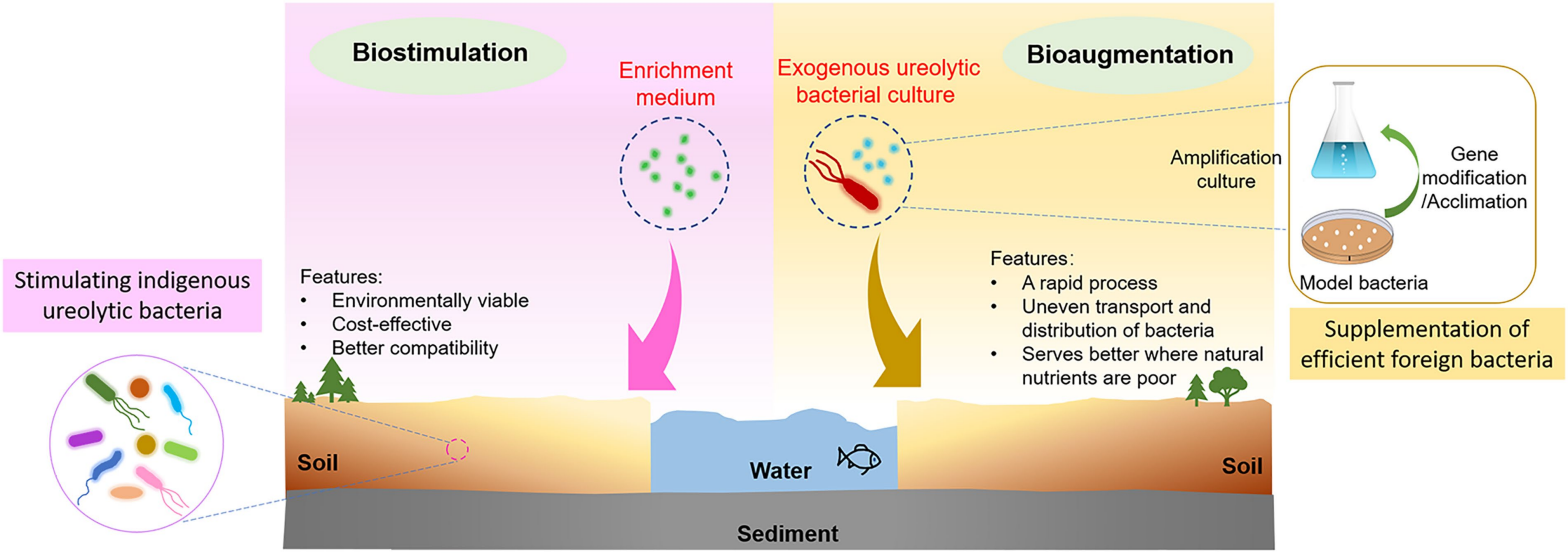
Pathways of MICP: bioaugmentation and biostimulation.

Bioaugmentation (supplementation of efficient foreign bacteria) or biostimulation (enrichment of the native bacterial population) are two typically used modes for MICP in HMs remediation (Fujita et al., 2008; Cuthbert et al., 2013; Gomez et al., 2017, 2019; Rahman and Singh, 2020). Bioaugmentation achieves MICP by introducing an exogenous and pure bacterial culture, such as the model bacterium *Sporosarcina pasteurii* (*S. pasteurii*; Graddy et al., 2018; Jiang et al., 2019). It is a quick procedure, which makes it quite appealing for a variety of applications, despite the high cost of cultivating and transporting bacterial cultures (Gomez et al., 2017). Chen et al. (2021a) used *S. pasteurii* to remediate Pb pollution in soil, and the results demonstrated that the amount of HMs leached from the soil decreased by 76.34% after remediation. Biostimulation achieves MICP by stimulating the growth of native microorganisms with desirable metabolic capabilities using an enrichment and nutrient medium (Gat et al., 2016). It is cost-effective and environmentally viable, since indigenous soil strains may have advantages in survivability and adaptability (Graddy et al., 2021), making them compatible with the applied polluted environment (Gat et al., 2016; Dhami et al., 2017; Graddy et al., 2018). For instance, the biostimulation process that applied to accelerate MICP in Cu immobilization also yielded favorable results; specifically, from 45.54 mg/kg, the exchangeable soluble fraction of Cu in soil fell to 1.55 mg/kg (Chen and Achal, 2019). The concept of bioaugmentation and biostimulation MICP biomineralization is illustrated in Figure 2.

**Figure 3:**
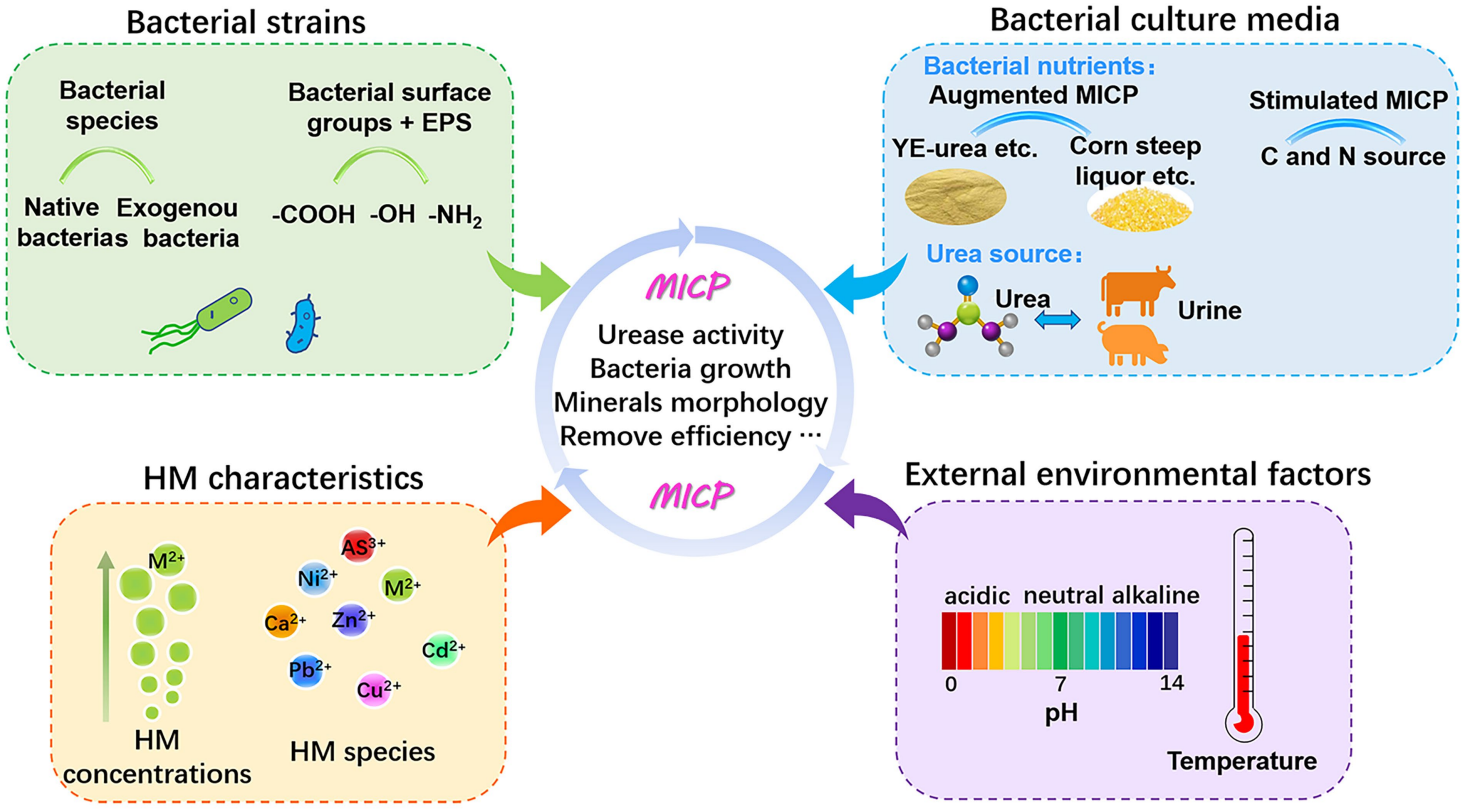
Key factors influencing the performance of MICP bioremediation.

To date, research on ureolytic MICP for metal removal has addressed the screening of bacteria with high urease activity and metal resistance, the morphology and property evaluation of precipitates, and the removal efficiency of various HMs (summarized in Supplementary Table S1, Supporting Information). Remediation efficiency relies on several factors like metal species, microorganisms (e.g., bacterial enzymatic properties and physiology), and microenvironmental conditions (e.g., pH, temperature, ion concentrations, and nutrient sources; as listed in Supplementary Table S1). All these factors govern the mobility and bioavailability of HMs for microbial transformation and biomineralization (Figure 3). Variations in these variables produce the major forces that govern the MICP process. Only by comprehending the regulatory mechanism can we utilize this technology more effectively.

### 3.1. Bacterial strains

The effectiveness of ureolytic MICP is strongly correlated with either the performance of exogenous causes or the performance of enriched native ureolytic bacteria. Bacteria’s capability to establish an alkaline nature through metabolism and subsequently precipitation has been identified as their key involvement in the process (Figure 1). A direct association exists between carbonate precipitation and urea quantity broken down by bacteria, determined by bacterial urease catalyst. Urease converts urea to products at least 10^14^ times faster than the rate of spontaneous decomposition (Wang et al., 2016). Thus, the quantity and activity of bacterial urease are crucial for the precipitation of bacterial carbonates. Numerous species of microorganisms exhibit urease activity at different level, thus leading to distinct MICP effects. The *Bacillus* group is a common type of bacteria used for urease production and precipitation (Kim and Lee, 2018; Jiang et al., 2019; Duarte-Nass et al., 2020; Chen et al., 2021a). For instance, with its ultra-high urease activity, *S. pasteurii*, a robust soil bacterium that can tolerate extreme alkaline environment, is the primary organism used for multiple applications, including remediation of HMs and radionuclides, biological self-healing concrete production, and soil improvement (Cuzman et al., 2015; Seifan and Berenjian, 2018; Chen et al., 2021a). Additionally, it should be noted from Supplementary Table S1 that a variety of HMs resistant indigenous ureolytic bacterial strains have been isolated from HM-contaminated environments and found to be effective for MICP under suitable conditions due to their high tolerance and adaptability to native environments.

Furthermore, bacterial surfaces play a vital function in the precipitation of calcium and HMs (Fortin et al., 1997; Wu et al., 2021). Diverse negative ion groups on the exterior surface and in the extracellular polymeric substances (EPS), such as hydroxyl, carboxyl, amine, and amide, can chelate positively charged metal ions (Braissant et al., 2007), which effectively reduce the free energy of crystal nucleation and favors heterogeneous nucleation (Dhami et al., 2013; Kim et al., 2017). Commonly, some bacterial cells can be completely embedded in growing carbonate crystals (Stocks-Fischer et al., 1999; Vekariya and Pitroda, 2013). The presence of bacterial cells could promote the formation of the thermodynamically unstable vaterite phase (Zhang et al., 2018). EPS can trap Ca^2+^ at specific pH, regulate crystallization, and impact the polymorphic development of CaCO_3_ crystals (Kawaguchi and Decho, 2002). Braissant et al. (2007) highlighted the potential role of EPS in the diversity of CaCO_3_ shapes. In the presence of glumanine, glutamic acid, and a significant quantity of EPS, vaterite can form fibro-radial spherulites (Chironi et al., 2003). Hence, bacterial cells and EPS can affect the polymorphism and morphology of minerals, which may affect the removal efficiency of HMs. Bai et al. (2021) revealed that Pb removal declined with the increasement of the vaterite faction within bio-precipitates. Bacterial cells and carbonate minerals also inevitably engage in adsorption, thereby accounting for the trap of HMs (Qiao et al., 2021).

In the case of HM remediation by ureolytic microorganisms, the diversity of microbial species led to the production of various HM-binding forms. *Raoultella planticola*, a ureolytic bacterial strain, transformed Pb^2+^ into light grey cerussite (PbCO_3_), whereas *Metschnikowia pulcherrima*, a ureolytic fungal strain, transformed Pb^2+^ into CaPbO_3_. Such instances were also disclosed by Hg^2+^ remediation (Eltarahony et al., 2021). Supplementary Table S1 also lists the final products of different representative HMs formed in MICP.

### 3.2. Bacterial culture media

#### 3.2.1. Bacterial nutrient

Because nutrients provide the necessary energy sources for bacterial growth and metabolic enzyme activity. Adequate nutrient availability is essential for the biomineralization of HMs. For the augmented MICP, bacteria are typically grown to the desired concentration in the laboratory using specific culture media. Some representative culture media are frequently selected for preparing target strains, including ammonium-yeast medium (Whiffin et al., 2007; Jiang et al., 2019; Liu et al., 2020), urea-yeast medium (Amiri and Bundur, 2018), urea rich-ammonium-yeast medium and nutrient broth-urea (NBU) medium (Zhang et al., 2018; Yi et al., 2021; Yin et al., 2021a; Li et al., 2022; see Supplementary Table S1). Either urea or ammonium can induce high urease activity in this situation. Carbon and nitrogen sources are among the most essential nutritional requirements, serving as sources of energy and heterotroph survival, respectively (Anderson and Jayaraman, 2003). According to the published works in Supplementary Table S1, yeast extract is the most common carbon source for ureolytic bacterial cultures. However, the consumption of yeast extract may result in an increase in cost, because its ratio to the total operating cost of urea-yeast medium is nearly 60% (Dhami et al., 2012). Alternative nutritional sources such as lactose mother liquor and corn steep liquor (CSL) that collected from agricultural or dairy industries have been used to significantly reduce the economic cost of cultivating (Achal et al., 2009; Dhami et al., 2012; Amiri and Bundur, 2018). Amiri and Bundur (2018) replaced yeast extract with CSL without compromising bacterial growth or precipitation capabilities. This substitution can not only reduce costs but also decrease the accumulation of environmental wastes through recycling, providing ample opportunities for MICP-based HMs removal on a large-scale.

For the stimulated MICP, appropriate enriched media (e.g., concentration and composition) for native ureolytic bacteria are the most important in ascertaining enrichment efficiency. Most ureolytic bacteria may be enriched efficiently supplemented with medium containing urea and/or ammonium (Wang et al., 2019; Park et al., 2022). Nevertheless, after simulation solution injection, indigenous bacterium may not attain the requisite ureolytic rate promptly. To maximize urease activity rapidly and increase ureolytic efficiency, the medium composition was systematically optimized by adding various carbon sources and nitrogen sources (organic or inorganic; Park et al., 2022). Wang et al. (2019) investigated the effect of enrichment medium type (yeast extract, malt extract and nutrient broth) on biostimulation efficiency. They discovered that more ideal enrichment medium are YE, NB, and other N-rich complex media, particularly the NBU medium that comprises a broad variety of amino acids, proteins, carbohydrates, vitamins, and minerals. Nonetheless, culture and enrichment effectiveness, the underlying expenses, and ecological dangers need to be stringently assessed and balanced carefully in large-scale field use.

#### 3.2.2. Urea source

For the urea source, a synthetic pure chemical, urea, is widely used in a number of MICP processes as the essential substance for ureolytic bacteria to produce carbonate precipitation. Despite this, multiple papers indicate that, during MICP bioremediation, substantial amounts of urea would cause excessive ammonium to enter soil or water, resulting in soil salinization, suppression of soil microbial biomass, and eutrophication of the water body (Dhami et al., 2013; Tian and Niu, 2015). This hinders the engineering application of MICP on a large scale. In addition, urea results from high-temperature and high-pressure mixing of ammonia and carbon dioxide by fossil fuel burning, leading to the release of greenhouse gases (Comadran-Casas et al., 2022). Thus, many scholars attempt to search environmentally friendly alternatives of urea, on the premise of ensuring the high efficiency of MICP. Fortunately, urine is considered a precious nutrient resource and is rich in urea [~1,000 mM urea in mammal urine (Yao et al., 2012)], which could be used to substitute the synthetic urea in MICP (Comadran-Casas et al., 2022). Chen et al. (2019) first used pig urine instead of industrial urea to produce CaCO_3_, by which increased bacterial growth in quartz-sand columns and decreased porosity over time. Bio-bricks were successfully manufactured *via* MICP using human urine as urea source, and exhibited well compressive strength (Lambert and Randall, 2019). Other natural substances, such as oyster shell wastes (OS), which is rich in calcium sources and nutrients like N\C and amino acids, could also be utilized by ureolytic bacteria, to achieve a similar Cd removal effect instead of urea (Peng et al., 2020).

### 3.3. HM characteristics

In general, the released free HMs may inhibit bacterial growth and urease activity by binding to the sulfhydryl groups of urease (Krajewska, 2008), thereby diminishing the efficacy of the treatment of targeted HMs (Chung et al., 2020; Chen et al., 2021b; Li et al., 2021b). The metal tolerance of a specific strain is a crucial parameter for an efficient ureolytic MICP process, which varies depending on the heavy metal species. For instance, minimum inhibitory concentration (MIC) of *S. pasteurii* exhibited different values for multiple metals (0.03–0.06 mM for Cd^2+^, 0.2–0.5 mM for Zn^2+^ and Cu^2+^, below 1 mM for Pb^2+^; Mugwar and Harbottle, 2016). Moreover, removal effectiveness of metal ions upon MICP changed with HM ions species and concentrations (Jain and Arnepalli, 2019; Kim et al., 2021; Qiao et al., 2021; Song et al., 2021), which also have a marked impact on the carbonate mineralogy, shape, and crystallinity of various precipitations. Kim et al. (2021) demonstrated that Pb and exhibited a high removal efficiency of greater than 99%, while Cu, Zn, and Cd exhibited a low removal efficiency of 30 ~ 60% at a low concentration of 0.05 mM or less. Along with an increase in initial HM ion concentration, the removal efficiency decreased, which can be attributed to increasing metal toxicity to cell growth and a lack of nucleation sites for the precipitation of metallic carbonates (Zhao et al., 2017; Peng et al., 2020). Bhattacharya et al. (2018) indicated that 98, 79, and 65% removal of Cd^2+^ was detected, with varying Cd concentrations of 5, 10, and 15 mg/L, respectively. Divalent metallic ions are generally removed through direct precipitation, where metal carbonate is formed, or by co-precipitation *via* substituting for Ca^2+^ within the carbonate phase, thereby decreasing their solubility and allowing sequestration for long periods (Achal et al., 2012; Kim et al., 2021). Multivalent ions, e.g., hexavalent chromium (Cr^6+^), may be reduced through co-precipitation of Cr with calcite and bio-reduction of the bacterial strain itself. By contrast, divalent metal ions have a more effective immobilization by MICP through directly precipitation (He et al., 2019). Sometimes, the presence of metal mixtures can increase or decrease metal toxicity and affect precipitation, thus more developments are needed in the area of MICP remediation for metal mixtures.

### 3.4. External environmental factors

External environmental factors, particularly pH and temperature, can have an effect on mineralization in terms of biological action, precipitation rate, and products properties. Ureolytic bacteria and urease perform well within their optimal pH or temperature ranges, as shown in Supplementary Table S1, but once these ranges are exceeded, their activities will diminish or cease entirely (Achal et al., 2011; Peng et al., 2020). The process of urea hydrolysis is temperature-dependent. In general, the optimal incubation temperature for biomineralization ranges from 20 to 37°C, and specific optimization selection based on other system conditions and medium concentration (Mitchell and Ferris, 2005). Mitchell and Ferris (2005) suggested that the urea-hydrolysis rate constants increased more than tenfold at 20°C compared to 15 and 10°C, no matter with or without Sr. At higher experimental temperatures, the proportion of Sr. incorporated into calcite increased dramatically. The optimal temperature of *Kocuria flava* (Achal et al., 2011) for copper removal was 30°C.

The pH plays a vital role for urea hydrolysis and mineral formation through affecting the urease activity and determining the acid-based chemical equilibria, thereby specifying the existence of carbonate and the precipitation processes (Kim et al., 2018; Omoregie et al., 2021). Based on the published cases, urease activity is optimal at a pH range of 6–10, but is significantly inhibited when the pH exceeds 10 or under extremely alkaline conditions (Stocks-Fischer et al., 1999; Torres-Aravena et al., 2018). Compared to acidic and alkaline conditions (pH 6.0 and 9.0, respectively), neutral to slightly alkaline conditions (pH 7.0–8.0) were more favorable for the removal of copper, demonstrating the optimal pH conditions of *Kocuria flava* for efficiently copper bioremediation (Achal et al., 2011). Some indigenous bacterium obtained from alkaline environments may have enhanced MICP performance under extreme alkaline conditions owing to their long-term adaptation process with gradual increased pH (Marin et al., 2021; Park et al., 2022). In MICP, urea hydrolysis causes a rapid rise in pH due to the release of ammonia, which may affect saturation states as well as the efficiency and extent of carbonate precipitation. Most calcium carbonate precipitation occurs at pH 8.5–9.5 (Stocks-Fischer et al., 1999; Lauchnor et al., 2013; Zhang et al., 2018), whereas carbonate tend to dissolve than precipitation at low pH levels (Seifan et al., 2017).

## 4. Strategies for enhancing the performance of ureolytic bacteria mediated HM remediation

Actually, HMs, such as Cu^2+^, Ni^2+^, Pb^2+^, Cd^2+^, Zn^2+^, Mn^2+^, can have negative effects on microbial growth through a variety of mechanisms, including breaking fatal enzymatic functions, functioning as redox catalysts in reactive oxygen species (ROS) generation, destroying ion control, and directly impacting protein and DNA synthesis (Medfu Tarekegn et al., 2020). For example, Cu^2+^ can trigger the generation of ROS by disrupting the bacterial respiratory chain, resulting in severe injury to DNA, protein and lipid peroxidation (Dupont et al., 2011). In HM contaminated sites, the living environment of microorganisms is quite stringent, and microbiostasis caused by the toxic effects of HMs or other contaminants is a significant barrier to the remediation (Song et al., 2021). Therefore, it is essential to develop innovative strategies for enhancing the tolerance of microbes to higher concentrations of pollutants and boosting their remediation effectiveness. Five categories of MICP bioremediation enhancement strategies are discussed in detail below.

### 4.1. Ureolytic microbial consortia

**Figure 4:**
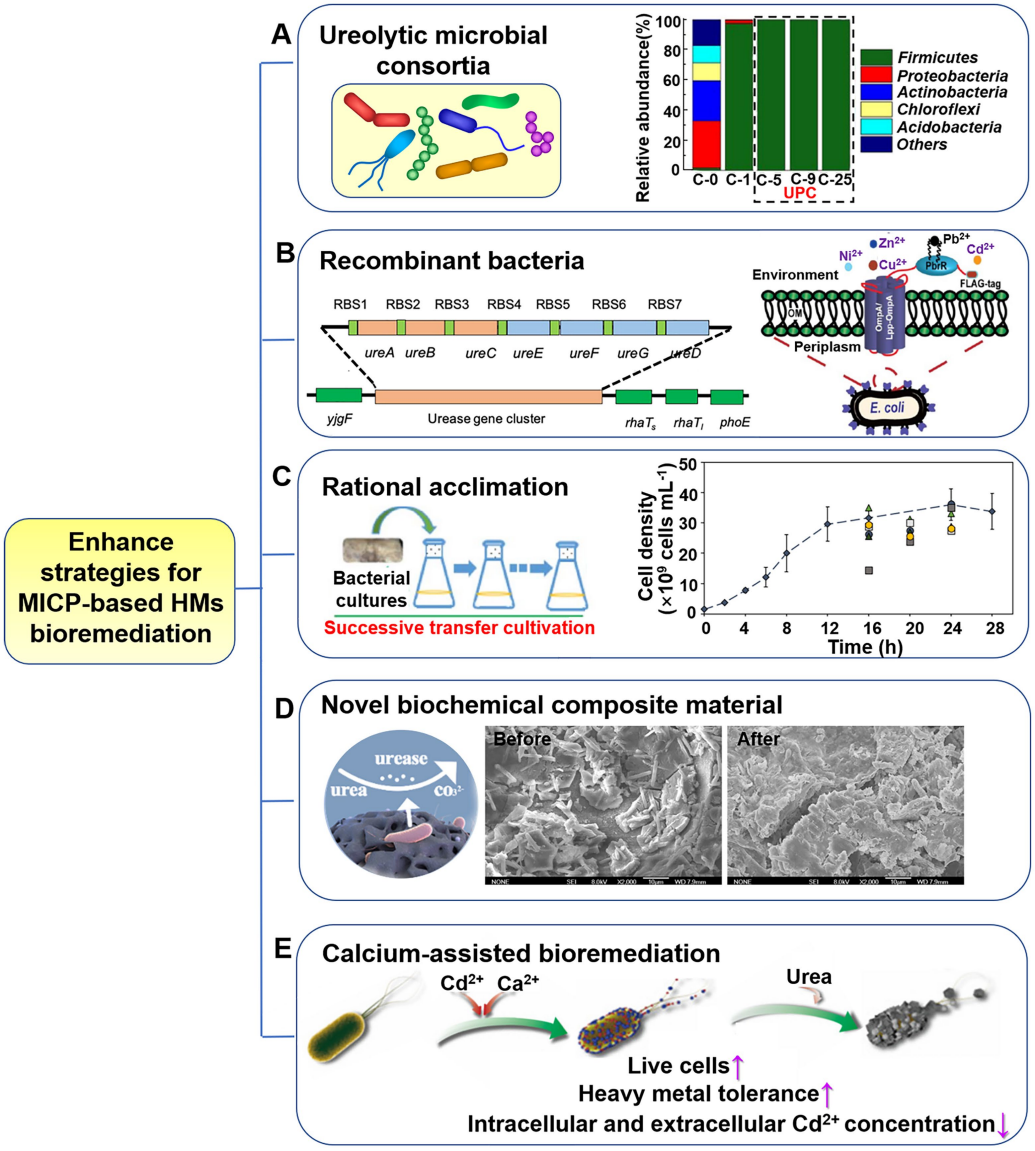
Enhancement strategies for ureolytic MICP-based heavy metals (HMs) bioremediation. **(A)** Ureolytic microbial consortia. Reproduced with permission from Yin et al. (2021a). **(B)** Recombinant bacteria. Reproduced with permission from Wei et al. (2014), Liang et al. (2018b). **(C)** Rational acclimation. Reproduced with permission from Oliveira et al. (2021) and Liang et al. (2018a). **(D)** Novel biochemical composite materials. Reproduced with permission from Song et al. (2021). **(E)** Calcium-assisted bioremediation. Reproduced from Fang et al. (2021).

The urea hydrolysis capacity and precipitation of HMs by bacterial isolates have been the primary focus of research on ureolytic MICP conducted with numerous bacterial isolates from a variety of environments (Bhattacharya et al., 2019; He et al., 2019; Cuaxinque-Flores et al., 2020). In such cases, it is recommended to use multi-component systems, such as microbial consortia, which is a more realistic depiction of the actual surroundings compared to single-component models. Compared to single strain, consortia composed of diverse microorganisms (as shown in Figure 4A) have higher adaptability and viability under harsh abiotic stresses, such as salinity, low or high temperatures, and other environmental extremes (e.g., HM contaminated sites; Kalyani et al., 2013; Ma et al., 2019; Bai et al., 2020; Marin et al., 2021). During the biomineralization treatment of hazardous contaminations, urease-positive microbial consortia also exhibit stable urease activity and can endure a broad variety of ecological stresses due to the synergistic impact of microorganisms in the group. Yin et al. (2021a) constructed a novel urease-producing consortium (UPC) *via* acclimation. The UPC exhibited high-efficiency Cd mineralization (93%; Figure 4A) and inhibited Cd releasing from sulfide tailings (Yin et al., 2021b). The bacterial community structure changed with different acclimation transfers (Figure 4A), resulting in a broad adaptability to a variety of environmental conditions: pH 4.0–11.0, temperature 10–45°C, and Cd concentration 0–200 mg/L. Of course, the existence of HMs will in return alter community composition and decrease the community diversity (Hu et al., 2021; Song et al., 2021; Yin et al., 2021a). The feasibility of Ca^2+^ and HMs removal from hypersaline produced water (PW) *via* MICP may cause complex challenges due to the distinct high salinity features of PW (ten-fold compared to seawater). Hu et al. (2021) constructed a stable ureolytic consortium with stronger hypersaline environmental adaptability and obtained significant Ca^2+^ removal efficiency (~96%), as well as organic contaminants (~100%), and HMs (~100% for As, Cd, Mn and Ni, 92.2% for Ba, 94.2% for Sr). Therefore, the introduction of bacterial consortium provides considerably higher urease activity, HM immobilization capacity than individual cultures, developing an efficient and economic method for HM bioremediation (Kang et al., 2015, 2016; Song et al., 2022).

Various microorganisms are capable of inducing carbonate precipitation through distinct biological pathways. MICP can be enhanced not only by bacterial consortia but also by co-culture of other microorganisms. Microalgal-bacterial consortia of *Chlorella* sp. and *S. pasteurii* induced biomineralization enhancement was also verified with relatively low-cost and significantly higher carbon sequestration efficiency (Xu et al., 2020). The synergetic action of microalgae and bacteria effectively enhanced the MICP performance, and simultaneous enhancement of biomass accumulation of *Chlorella* sp. was executed by *S. pasteurii*. Hence, in view of the advantage of synergistic microbial consortia, the construction and modification of ureolytic microbial consortia with strong adaptability to different environmental conditions are worthy of further exploration. This may provide novel microbial resource for HM biomineralization.

### 4.2. Recombinant bacteria

Due to the complexity of environmental conditions, efficient *in-situ* HMs bioremediation requires microorganisms that can withstand the synergistic toxicity of multiple HM ions, variations in hydrology, and diverse combinations of contaminants. With the rapid development of biotechnology (especially molecular biology), genetically engineered strain is a good choice to enhance the survivability and adaptability of microbes in volatile situations, which may hold great potential for substantially improving MICP performance. The successful expression of urease-producing genes from *S. pasteurii* in *Escherichia coli* (*E. coli*) demonstrates the viability of using engineered strains for MICP (Liang et al., 2018b; Qiu et al., 2021; Figure 4B, left). The rationally designed strains demonstrated the potential to regulate biogenic CaCO_3_ precipitation by tailoring the size and shape of CaCO_3_, exhibiting distinctive morphology and nanomechanical properties. Future study in this field needs focus on design, development, and usage of genetically engineered and high urease-producing bacteria, alongside more significant ecological adaptation, and enhanced HM resistance for the application of MICP in bioremediation. Recombinant *E. coli* with overexpressing of a phytochelatin synthase gene (PcPCS1) from bean pear was shown to have markedly increased tolerance of cadmium, copper, sodium, and mercury in comparison to the non-engineered *E. coli* (Li et al., 2015). Additionally, the detection and quantification of HMs with extremely low concentration are also critical issues in HM-contaminated sites. Consideration will also be given to the multifunctional microorganism system that can simultaneously realize efficient remediation and detection of HM pollution. For instance, a surface-engineered *E. coli* that can selectively remediate and detect lead in the environment was designed, which harbored lead-specific binding protein (PBrR) and the promoter region for PbrR from *Cupriavidus metallidurans* CH34 along with the red fluorescence protein (Wei et al., 2014; Figure 4B, right). The application of designed genetic engineering microbes in ureolytic MICP is in its infancy, but it holds great promise for future. In actual environmental applications, however, the possibility of biosafety concerns resulting from the environmental escape of engineered bacteria must be considered, and risk response measures must be formulated in accordance with design principles and applicable laws and regulations.

### 4.3. Rational acclimation

Acclimation is a common phenotypic response to environmental changes, which usually refers to artificial selection and breeding of wild species to obtain dominant strains that have tolerance to extreme environments and meet human or industrial requirements (Steensels et al., 2019). Through the acclimation process, microbes gained the capacity to efficiently degrade particular substance, cope with specific stress factors and produce desirable compounds (Liu et al., 2013; Madigou et al., 2016; Hou et al., 2020). In the application of MICP technology in environmental remediation, there are high requirements for the characteristics of bacteria (Mwandira et al., 2019; Do et al., 2020; Choi et al., 2021), such as toxic metal, alkaline, and psychro-tolerances. Therefore, it is necessary to screen for highly tolerant bacterial strains. In this case, acclimated bacteria are preferred due to the belief that acclimation enhances performance and is therefore adaptive. Bacteria used in toxic metal remediation can adapt to high concentrations of HMs *via* multifarious mechanisms for resistance, such as intra/extra-cellular sequestration, efflux mechanism, permeability barrier, and the creation of resistant populations through metabolic and molecular changes (Kepenek et al., 2020).

An acclimation strategy, consisting in a regular step-wise adaptation of the target strains to stressful condition was commonly employed. Bacteria can be gradually exposed to concentration gradients of toxic HMs through successive transfer cultivation and enhanced adaptability to the contaminated environment during the acclimation process (Oliveira et al., 2021; Yin et al., 2021a). Oliveira et al. (2021) investigated copper toxicity on *S. pasteurii* and found that the MIC reported here is significantly lower than the copper concentration of mine tailings used in this study. So, increasing the bacterial resistance to stressed copper-containing conditions is the guarantee of successful application here. A pre-emptively acclimation was performed as follows (from 50 to 600 mg/L copper): 10 ml of a *S. pasteurii* culture was inoculated into 90 ml of medium containing 50 mg/L copper ions for 24 h. Ten ml of this acclimatized culture was then subcultured into 90 ml of fresh medium containing 50 mg/L copper ions and incubated for 24 h. This procedure was repeated in the following day, in order to further screen copper tolerant cells and outcompete the other cells. Next, concentration of copper was increased to 100 mg/L and repeated acclimation process was carried out as above. The results shown that the acclimated culture performed well in each increased copper concentration, even up to 600 mg/L, indicating *S. pasteurii* that can tolerant higher copper concentrations was successfully acclimated (as shown in Figure 4C). In addition to this acclimation of typical ureolytic bacterium, there is also acclimation to construct urease-positive bacterial consortium through multi-acclimation transfers (refer to Section 4.1). Besides, many microorganisms exposed to the contaminated environments would acclimate themselves to adapt to their existing conditions. Bacteria may experience acute or gradual HM acclimation in their habitats, depending on their proximity to the source of metal pollution. Special bacteria (e.g., urease-producing strains) that can tolerate HMs from these polluted environments, such as mine wastewater, tailings, polluted soil, and sludge, are typically screened and isolated (Supplementary Table S1). Subsequently, they may be identified according to molecular characterization and get involved in bioremediation.

With the advancement of modern microbial technology, the acclimated microorganisms have greater specificity and efficacy in removing HMs, indicating a brighter future for the use of microorganisms in environmental remediation. However, the maintenance of the resistance to the HMs after the acclimation will have to be assessed. Moreover, the acclimation process is usually not instantaneous and still time-consuming. Therefore, novel acclimation strategies must be developed to compensate for these deficiencies.

### 4.4. Novel biochemical composite materials

Desired metabolic activity and survivability for bacteria in extreme environments are crucial for its wide applications. In bioaugmentation, microbial cultures to be transferred to the contaminated sites are thriving cell suspensions produced under optimum conditions. However, in real cases, the microbial inoculants decreased shortly after injection owing to the exposure to the complex environmental stresses, such as temperature, water, pH, nutrients and toxic pollutants (Tyagi et al., 2011). For successful bioremediation, the adaptability of the inoculated cultures is of the utmost importance. The immobilization of microbial cells on carrier materials provides a number of benefits, including increased urea hydrolysis rates and decreased contamination risks. This can be accomplished through a variety of physical and chemical techniques, such as encapsulation, adsorption, covalent bonding, and other mechanisms. The use of carrier materials can provide microbes with physical support and improved access to nutrients, moisture, and oxygen, resulting in a higher survival rate under harsh conditions. Li et al. (2022) prepared a sustainable material carrier, corncob powder, for the immobilization of ureolytic bacterium; this treatment sustains their survivability in extremely Cd-contaminated soils and boosts their functionality for Cd remediation. The porous structure of corncob provided a high surface area for the entrapment of cells, and its high organic matter content could support nutrients for the ureolytic strain, along with low cost render it suitable candidate as a carrier.

Seeking for suitable enhancer with MICP is a proven method for more efficient HMs removal. Studies have shown that materials adsorption methods and biomineralization were frequently used alone for the HMs remediation in polluted area and presented their respective benefits and drawbacks (Liu and Lian, 2019; Xue et al., 2022). Adsorption has become one of the most commonly used methods for HM treatments recently due to its inexpensiveness and high efficiency; however, the durability of adsorbents must be considered. To give full play to the strengths of the adsorbents and MICP method for HMs removal and make the two methods complementation, a novel microbial adsorbent, “Scoria@UF1,” was developed by loading urease microflora on the scoria material (Song et al., 2021; Figure 4D). The combination of scoria adsorption and urease microbial mineralization was revealed a high-efficient remediation process, and the two ways were continuous and non-overlapping in terms of adsorption time. Functional groups on the surface of scoria contribute to the adsorption *via* the formation of chemical bonds with Pb^2+^ and Cd^2+^. HM ions are then precipitated in the form of carbonate minerals as the urease concentration increases in the reaction substrate. When biological fixation of HM ions on Scoria@UF1 tends to saturate and microbial activity gradually declines, the adsorption of HMs reaches equilibrium.

Biochar is another economically alternative sorbent to enhance the removal efficiency of HMs due to their porous structure and abundant surface functional groups (Zhang et al., 2017). Zhang et al. (2019) hypothesized that biochar maybe favorable for the enhancement of MICP-based HMs immobilization. Surprisingly, biochar inhibited the urease-producing bacterium *Bacillus cereus* NS4-induced calcite and suppressed the Ni remediation (remove efficiency decreased from 89 to 74%). This is owing to the encapsulation of bacterial cells within the pores of biochar and the resulting aggregates blocking urease access and possible toxic substances in biochar. Various biochars derived from other biomass wastes should be further evaluated. Nevertheless, biochars as carrier material of microorganisms were successfully used, for example, to load a plant growth promotion bacterium (PGPB) strain (Ma et al., 2020). With the treatment of this biochemical composites material by combining biochar with bacteria, ryegrass biomass was significantly increased by 77.8% and its Cd concentration decreased by 48.5%. Therefore, the immobilization carrier may need to be tailored to each application, and additional research could be conducted to optimize the properties of existing carrier materials and seek out novel carriers. With the variety of immobilization procedures and carriers available, MICP technology may be advanced greatly using immobilization.

### 4.5. Calcium-assisted bioremediation

Calcium carbonates have long been used as scavengers of HMs by efficient adsorption (Liu and Lian, 2019; Bai et al., 2021) or by incorporating divalent HM cations in calcium carbonate crystals (Bhattacharya et al., 2018; Zhang et al., 2022). This is crucial for the application of MICP in metal immobilization technology. Therefore, the introduction of active Ca^2+^ to the bioremediation process is a straightforward method for alleviation of high HM toxicity, protecting ureolytic bacteria against HM stress and elevating the remediation performance (Bai et al., 2021; Fang et al., 2021; Kim et al., 2021; Xue et al., 2022; Zhang et al., 2022;). The researchers discovered Ca^2+^ can compete with Cd^2+^ for surface binding and transport into the cells, therefore significantly enhanced the Cd^2+^ resistance of ureolytic bacterium (Fang et al., 2021; Figure 4E). The presence of Ca inhibited biosorption and bioaccumulation of Cd^2+^ in *S. pasteurii* cells, enabling an obvious enhancement in Cd-tolerance (a 20-fold improvement), survivability, and urease activity of *S. pasteurii* under serious Cd stress. Similar improvement was also verified in bacteria-based MICP of Cr with the addition of Ca (Zhang et al., 2022). Nevertheless, Ca-mediated promotion effect on the MICP-based bioremediation process presented the likelihood of selectivity of target metals. When exposed to Cu^2+^ and Zn^2+^ stress, the supplement of Ca could promote bacteria growth of up to about 70-folds and also significantly enhance urease activity. In contrast, the improvement effect of Ca is less pronounced for Mn^2+^ and Ni^2+^ (Fang et al., 2021; Zhang et al., 2022). The calcium source is found to remarkably influence HM immobilization efficiency by changing the initial precipitation phases. Jiang et al. (2019) determined a higher Pb removal percentage in the case of calcium chloride as calcium source as compared to calcium acetate. Besides Ca^2+^, there are also other chemicals that can influence bacterial growth and urease activity. Higher urea concentrations, for instance, can enhance copper tolerance of bacteria, exhibiting a positive correlation between them (Duarte-Nass et al., 2020).

### 4.6. Other approaches

In addition to these strategies as mentioned above to improve the bacterial tolerance to HMs, adjusting biomineralization program to reduce the toxic effect of HMs on bacteria was also an efficient strategy. In *in situ* MICP process to resolve cadmium releasing issue from sulfide tailings, Yin et al. (2021b) adopted 2-phase injection biomineralization program, which began with the injection of abundant cultures with a certain retention time followed by the injection of mineralization solution with optimum concentration and input rate. And repeated injection for the circularly mineralization improved the Cd immobilization efficiency and uniformity in the tailings (Cd content fell by 80.7% after 7 cycles of mineralization). Bhattacharya et al. (2018) conducted an *in vitro* study utilizing urease-containing cell-free supernatant of cultures with high urease levels (1,156 U/ml) to trigger remediation of Cd^2+^. This kind of urease-aided carbonate mineralization is more straightforward and can function even in HMs contaminated environments with limited ureolytic bacterial growth. Bacteria can form HM resistance mechanisms by regulating their physiological and biochemical states to combat with HMs. During biocalcification, the surface-layer protein (Slp) of ureolytic bacteria, repeated protein monomers that self-assembled into ordered structures, also plays an essential role in the HM immobilization mechanism. Li et al. (2021b) reported a positive correlation between metal concentrations and the Slp. This biological compounds not only can execute biosorption, but also can improve survivability of bacteria in harsh HM-contaminated environments (Suhr et al., 2014; Li et al., 2021b). More and more enhancement strategies may be proposed that lead to MICP mediated pollutant remediation more efficiently, cheaper, more technically feasible and environmentally friendly, laying the groundwork for future large-scale *in situ* application.

## 5. Challenges and future perspectives

After decades of development, MICP technology is now widely applied in the remediation of HM pollution, which has the advantages of high efficiency, low cost and environmental friendliness. Nevertheless, there are still many bottlenecks and actual obstacles for widespread application, especially for *in situ* bioremediation. A successful implement in controlled lab conditions does not guarantee similar success in a complex real-case setting. In fact, intensive laboratory research can shed light on the relationship between microbes and environmental factors, guiding on-site interventions for decontamination and bridging the gap between lab-scale and field-scale bioremediation.

Several fundamental issues pertaining to MICP bioremediation mechanisms remain to be resolved:

1. *Strengthen the study on the inner relationship of bacterial resistance system and their HM remediation ability.* Bacterial resistance mechanisms to HMs exhibit specificity on the basis of bacterial species and HMs types. Active toxic HMs influence the diversity and metabolic activity of microorganisms and in turn microorganisms can develop resistance systems to overcome the stress from HMs. To enhance the bioremediation performance of microorganisms, the molecular mechanism of HM detoxification needs to be further elucidated.
2. *Integrated multi-technology.* Various physicochemical methods have been used for HM removal, and the combination of different techniques is the general trend for large-scale applications in real situation, which is an effective way to save cost and improve efficiency. For example, as mentioned in Section 4.4, some novel materials can be selected to provide an optimized environment for microbes (as carrier or nutrients, etc.) or be used as adsorbent materials that synergistic remediation with repair role in ureolytic mineralization. It also becomes more important to explore the possibility of using existing *in situ* materials to facilitate the MICP process and lower the operational cost.
3. *Intensify the analysis by computer simulations.* Numerical models and programs were presented to design and implement for comprehending the MICP processes and predesigning lab and field experiments. Ideally, computer simulations can be carried out in advance of the field application to guide the parameters design (Qin et al., 2016; Cunningham et al., 2018). Also, computer programs can accurately predict and calculate elemental speciation, distribution and ion activity in various aqueous media, such as Visual MINTEQ software (Jiang et al., 2019; Duarte-Nass et al., 2020; Xue et al., 2022). This offers the ease and simplicity for theoretical interpretation of HMs removal through ureolytic MICP, which is not available from analytical techniques, enhancing our understanding on the mechanisms of HMs removal. Further research is needed to systematically develop the simulation-aided MICP technique.
4. *Pay attention to incidental environmental concern.* Ammonium production is the main environmental concern because of the addition of a large amount of urea from MICP. Seeking effective substitute for commercially available urea (such as oyster shell wastes (Peng et al., 2020) or recovering ammonium in the MICP treated system (e.g., recovered as a fertilizer (NH_4_)_2_SO_4_; Hu et al., 2021) are effective and eco-sustainable strategies for the large-scale biomineralization applications. In MICP-treated sites, phosphoric acid was introduced to make the wastewater neutral to overcome the high pH caused by MICP, thereby enhancing soil fertility and plant growth when used for irrigation (Song et al., 2022). Nevertheless, the released ammonia could be helpful in bioremediation of HMs, such as the facilitated co-precipitation of arsenic by forming gwihabaite (Achal et al., 2012). Detailed investigation and comprehensive understand on these problems as well as processing strategies will be required for long-term applications.

## 6. Conclusion

Microbially induced carbonate precipitation is a biogeochemical process, which has been incorporated into environmental technologies for fracture remediation, corrosion prevention of building materials, and toxic HMs sequestration. This paper reviews the recent progress of MICP in efficient and environmentally friendly bioremediation of HMs. Its execution mainly refers to two pathways of biostimulation or bioaugmentation, by enrichment of indigenous ureolytic bacteria and injection of exogenous bacterial cultures, respectively, guiding the employment of suitable methods in actual environments. Multiple factors such as pH, temperature, bioavailability of nutrient, toxic substances level, and indigenous microbial population influence remediation strategies in adverse contaminated environments. Specially, the HM tolerance of microorganisms is crucial for growth and urease activity, determining the feasibility and success of MICP treatment in HM remediation. To enhance the bioremediation efficiency by MICP, several broad strategies have been implemented: using ureolytic microbial consortia, genetically engineered bacteria, rational acclimation, fabricating novel biochemical composite materials, calcium-assisted bioremediation. There should be a focus on acquire bacteria with high enzyme-producing ability, high environmental adaptability, and high efficiency in bioremediation. Future studies will evaluate the bacterial tolerance and environmental factors and feasibility in actual systems, ultimately to optimize for field-scale applications. Numerous innovations in different techniques and integration of computer simulation are recommended, which may provide us a deeper understanding on MICP bioremediation mechanism and attain better ways to deal with various environmental problems. We believe that research in this area will continue to grow swiftly in the coming, which may bring environmental benefits along with economic and social benefits.

## Author contributions

WZ: conceptualization, methodology, writing—original draft, writing—reviewing and editing; HZ: writing—review and editing; RX and HL: review and editing; HQ: help for creating figures; KZ: conceptualization, methodology, writing and editing, and reviewing the entire article. All authors contributed to the article and approved the submitted version.

## Funding

This work was supported by the National Natural Science Foundation of China (22006110), the Natural Science Foundation of Jiangsu Province of China (BK20200987), and the National Key R&D Program of China (2018YFA0902102).

## Conflict of interest

The authors declare that the research was conducted in the absence of any commercial or financial relationships that could be construed as a potential conflict of interest.

## Publisher’s note

All claims expressed in this article are solely those of the authors and do not necessarily represent those of their affiliated organizations, or those of the publisher, the editors and the reviewers. Any product that may be evaluated in this article, or claim that may be made by its manufacturer, is not guaranteed or endorsed by the publisher.
