# Supplementary material for: Heavy metal bioremediation using microbially induced carbonate precipitation: Key factors and enhancement strategies
Source: Front Microbiol. 2023 Feb 2;14:1116970. doi: 10.3389/fmicb.2023.1116970 (PMC9932936; doi:10.3389/fmicb.2023.1116970)
Supplement: Supplementary file 1 [file Table_1.docx]

Supplementary Material

Table S1. Summary of assay conditions for HM sequestration through the ureolytic MICP process.

| Bacteria strain | Source | Medium | Ca^2+^/mM | Urea/g/L | Tem./℃ | Initial pH | HM species | HM tolerance | Maximum removal rate | Urease activity | Removal mechanisms/ products | Refs |
| --- | --- | --- | --- | --- | --- | --- | --- | --- | --- | --- | --- | --- |
| *Sporosarcina pasteurii* | ATCC 6452 | NH_4_-YE medium | 200 | 12 | 30 | — | Pb^2+^ | 50mM | 95% | 10 mM urea/min | PbCO_3_ and CaCO_3_ | (Jiang et al., 2019) |
| *Sporosarcina pasteurii* | KCTC 3558 | yeast extract, proteose peptone, glucose, NaCl | 30 | 20 | 25 | 7 | Pb^2+^, Sr^2+^ | — | ＞99% | — | Co-precipitation | (Kim et al., 2021) |
| *Sporosarcina pasteurii* | KCTC 3558 | Tryptic soy broth (TSB) | 450 | 27 | 25 | 7.0 | Cu^2+^ | 0.39 mg/L | — | — | — | (Chung et al., 2020) |
| *Sporosarcina pasteurii* | CCCT 16.12 | beef extract, peptone, yeast extract, NaCl | 0 | 20 | 30 | 7 | Cu^2+^ | 0.5 mM | — | — | Copper carbonates: Cu_2_CO_3_(OH)_2_, Cu_3_(CO_3_)_2_(OH)_2_ | (Duarte-Nass et al., 2020) |
| *Sporosarcina pasteurii* | ATCC 11859 | Casein peptone, soya peptone, NaCl | 1000 | 20 | — | 7.3 | Pb^2+^ | — | 76.3% | — | — | (Chen et al., 2021a) |
| *Sporosarcina ginsengisoli* | Contaminated soil | NB | 25 | 20 | 30 | 8.0 | As^3+^ | 50 mM | 96.3% | 412 U/mL | Incorporated in calcite with CO_3_^2-^ being replaced by HAsO_3_^2-^ | (Achal et al., 2012) |
| *Sporosarcina pasteurii* | CGMCC | YE | 50 | 60 | 28 | 9.2-9.3 | Cd^2+^ | 5620.5 mg/L | 99.6% | 4–5 U/mL | CdCO_3_, CaCO_3_, and (Ca0.67, Cd0.33)CO_3_ | (Fang et al., 2021) |
| *Serratia marcescens* | NCIM 2919 | Peptone, NaCl, Yeast extract, Beef extract | 25 | 20 | 30 | 7.5 | Cd^2+^ | 25 mg/L | 96% | 1156 U/ mL | Co-precipitation of Cd^2+^ and Ca^2+^ | (Bhattacharya et al., 2018) |
| Urease bacteria group (UF1) | Activated sludge soil | KH_2_PO_3_, NaCl, glucose, trace element | 0 | 20 | 25 | 6.8 | Pb^2+^ | 1 mM | — | 6 mM/(L･min) | Scoria adsorption and urease microbial mineralization | (Song et al., 2021) |
| *Bacillus* sp. WA | Metal polluted soil | NBU | 50 | 20 | 30 | 8.0 | Cd^2+^ | 50 mg/L | 68.5% | ~450 U/mL | CdCO_3_ | (Li et al., 2022) |
| Urease-producing consortium (UPC) | Copper mine | NBU | 25 | 30 | 30 | natural pH | Cd^2+^ | 100 mg/L | 92.9% | 18.4-22.5 mM/min | (Ca0.67, Cd0.33)CO_3_ phase | (Yin et al., 2021a) |
| UPC | Copper Mine | NBU | 330 | 20 | 25 ± 3 | natural pH | Cd^2+^ | 3 μg/L | 80.7% | — | — | (Yin et al., 2021b) |
| UPC | Activated sludge | NB | 120 | 4 | ~20 | — | Ca^2+^ | — | ~96% | — | — | (Hu et al., 2021) |
|  |  |  |  |  |  |  | As^3+^ |  | 92.2% |  |  |  |
| *Brevundimonas diminuta* | Mines polluted soil | LB | 4.5 | 10 | 30 | 7 | Cd^2+^ | 200 mg/L | 99.2% | — | CdCO_3_/Cd_3_(PO_4_)_2_ | (Ali et al., 2021) |
|  |  |  |  |  |  |  | Zn^2+^ |  | 99.7% |  | ZnCO_3_/ZnHPO_4_/Zn_2_(OH)PO_4_/Zn_3_(PO_4_)_2_ |  |
| *Lysinibacillus* sp. GY3 | E-waste area | NBU | 50 | 20 | 37 | 8.0 | Cu^2+^ | 0.1 g/L | 98.7% | 50 U/mL | — | (Li et al., 2021b) |
|  |  |  |  |  |  |  | Pb^2+^ | 1 g/L | 98.8% | 32 U/mL |  |  |
| *Bacillus* sp. GZ-22 | Mining soil | LB | 0 | 133.4 | 35 | 6 | Cd^2+^ | 250 mg/L | 60.7 % | 0.83 mM/min | CdCO_3_ and adsorption | (Zhao et al., 2017) |
| *Raoultella planticola* | From 50 isolates | NB | 180 | 20 | 30 | 7.2 | Pb^2+^ | 0.35 g/L | Complete removal | 639 U/mL | PbCO_3_ | (Eltarahony et al., 2021) |
|  |  |  |  |  |  |  | Hg^2+^ |  |  |  | HgO_2_ |  |
| *Enterobacter* sp. | Mining sediment | LB | 40 | 20 | 37 | 4-9 | Cd^2+^ | 100 mg/L | 99.5 % | — | — | (Peng et al., 2020) |
| *Bacillus cereus* | Nickel mining | NB | 0 | 15 | 10 | 5-11 | Ni^2+^ | 400 mg/L | 73.5% | 194.6 U/mL | Ni_2_CO_3_(OH)_2_·H_2_O | (Do et al., 2020) |
| *Staphylococcus epidermidis* | Industrial area | NB | 25 | 20 | 30 | 8.2  7.7 | Pb^2+^ | 50 mg/L | 86% | 25 U/mL | — | (He et al., 2019) |
|  |  |  |  |  |  |  | Cr^6+^ |  | 76.8% | 8 U/mL |  |  |

## References

Achal, V., Pan, X., Fu, Q., Zhang, D. (2012). Biomineralization based remediation of As (III) contaminated soil by *Sporosarcina ginsengisoli*. *J. Hazard. Mater.* 201, 178-184. doi: 10.1016/j.jhazmat.2011.11.067

Ali, A., Li, M., Su, J., Li, Y., Wang, Z., Bai, Y., Ali, E. F., Shaheen, S. M. (2021). *Brevundimonas diminuta* isolated from mines polluted soil immobilized cadmium (Cd^2+^) and zinc (Zn^2+^) through calcium carbonate precipitation: Microscopic and spectroscopic investigations. *Sci. Total Environ.* 813, 152668. doi: 10.1016/j.scitotenv.2021.152668

Bhattacharya, A., Naik, S. N., Khare, S. K. (2018). Harnessing the bio-mineralization ability of urease producing *Serratia marcescens* and *Enterobacter cloacae* EMB19 for remediation of heavy metal cadmium (II). *J. Environ. Manage.* 215, 143-152. doi: 10.1016/j.jenvman.2018.03.055

Chen, M., Li, Y., Jiang, X., Zhao, D., Liu, X., Zhou, J., He, Z., Zheng, C., Pan, X. (2021a). Study on soil physical structure after the bioremediation of Pb pollution using microbial-induced carbonate precipitation methodology. *J. Hazard. Mater.*  411, 125103. doi: 10.1016/j.jhazmat.2021.125103

Chung, H., Kim, S. H., Nam, K. (2020). Inhibition of urea hydrolysis by free Cu concentration of soil solution in microbially induced calcium carbonate precipitation. *Sci. Total Environ.* 740, 140194. doi: 10.1016/j.scitotenv.2020.140194

Do, H., Wang, Y., Long, Z., Ketehouli, T., Li, X., Zhao, Z., Li, M. (2020). A psychrotolerant Ni-resistant *Bacillus cereus* D2 induces carbonate precipitation of nickel at low temperature. *Ecotoxicol. Environ. Saf.* 198, 110672. doi: 10.1016/j.ecoenv.2020.110672

Duarte-Nass, C., Rebolledo, K., Valenzuela, T., Kopp, M., Jeison, D., Rivas, M., Azócar, L., Torres-Aravena, Á., Ciudad, G. (2020). Application of microbe-induced carbonate precipitation for copper removal from copper-enriched waters: Challenges to future industrial application. *J. Environ. Manage.* 256, 109938. doi: 10.1016/j.jenvman.2019.109938

Eltarahony, M., Kamal, A., Zaki, S., Abd‐El‐Haleem, D. (2021). Heavy metals bioremediation and water softening using ureolytic strains *Metschnikowia pulcherrima* and *Raoultella planticola*. *J. Chem. Technol. Biotechnol.* 96, 3152-3165. doi: 10.1002/jctb.6868

Fang, L., Niu, Q., Cheng, L., Jiang, J., Yu, Y.-Y., Chu, J., Achal, V., You, T. (2021). Ca-mediated alleviation of Cd^2+^ induced toxicity and improved Cd^2+^ biomineralization by *Sporosarcina pasteurii*. *Sci. Total Environ.* 787, 147627. doi: 10.1016/j.scitotenv.2021.147627

He, J., Chen, X., Zhang, Q., Achal, V. (2019). More effective immobilization of divalent lead than hexavalent chromium through carbonate mineralization by *Staphylococcus epidermidis* HJ2. *Int. Biodeterior. Biodegrad.* 140, 67-71. doi: 10.1016/j.ibiod.2019.03.012

Hu, L., Wang, H., Xu, P., Zhang, Y. (2021). Biomineralization of hypersaline produced water using microbially induced calcite precipitation. *Water Res.* 190, 116753. doi: 10.1016/j.watres.2020.116753

Jiang, N. J., Liu, R., Du, Y. J., Bi, Y. Z. (2019). Microbial induced carbonate precipitation for immobilizing Pb contaminants: Toxic effects on bacterial activity and immobilization efficiency. *Sci. Total. Environ.* 672, 722-731. doi: 10.1016/j.scitotenv.2019.03.294

Kim, Y., Kwon, S., Roh, Y. (2021). Effect of divalent cations (Cu, Zn, Pb, Cd, and Sr) on microbially induced calcium carbonate precipitation and mineralogical properties. *Front. Microbiol.* 12, 763. doi: 10.3389/fmicb.2021.646748

Li, W., Fishman, A., Achal, V. (2021b). Ureolytic bacteria from electronic waste area, their biological robustness against potentially toxic elements and underlying mechanisms. *J. Environ. Manage.* 289, 112517. doi: 10.1016/j.jenvman.2021.112517

Li, W., Yang, Y., Achal, V. (2022). Biochemical composite material using corncob powder as a carrier material for ureolytic bacteria in soil cadmium immobilization. *Sci. Total. Environ.* 802, 149802. doi: 10.1016/j.scitotenv.2021.149802

Peng, D., Qiao, S., Luo, Y., Ma, H., Zhang, L., Hou, S., Wu, B., Xu, H. (2020). Performance of microbial induced carbonate precipitation for immobilizing Cd in water and soil. *J. Hazard. Mater.* 400, 123116. doi: 10.1016/j.jhazmat.2020.123116

Song, H., Wang, C., Kumar, A., Ding, Y., Li, S., Bai, X., Liu, T., Wang, J., Zhang, Y. (2021). Removal of Pb^2+^ and Cd^2+^ from contaminated water using novel microbial material (Scoria@ UF1). *J. Environ. Chem. Eng.* 9, 106495. doi: 10.1016/j.jece.2021.106495

Yin, T., Lin, H., Dong, Y., Li, B., He, Y., Liu, C., Chen, X. (2021a). A novel constructed carbonate-mineralized functional bacterial consortium for high-efficiency cadmium biomineralization. *J. Hazard. Mater.* 401, 123269. doi: 10.1016/j.jhazmat.2020.123269

Yin, T., Lin, H., Dong, Y., Wei, Z., Li, B., Liu, C., Chen, X. (2021b). Inhibition of cadmium releasing from sulfide tailings into the environment by carbonate-mineralized bacteria. *J. Hazard. Mater.* 419, 126479. doi: 10.1016/j.jhazmat.2021.126479

Zhao, Y., Yao, J., Yuan, Z., Wang, T., Zhang, Y., Wang, F. (2017). Bioremediation of Cd by strain GZ-22 isolated from mine soil based on biosorption and microbially induced carbonate precipitation. *Environ. Sci. Pollut. Res. Int.* 24, 372-380. doi: 10.1007/s11356-016-7810-y
